# Health Literacy, Beliefs About Medication, and Medication Adherence in Patients With Multiple Sclerosis in Jordan: A Cross‐Sectional Study

**DOI:** 10.1002/hsr2.71672

**Published:** 2025-12-18

**Authors:** Walid Al‐Qerem, Sawsan Khdair, Dunia Basem, Anan Jarab, Judith Eberhardt

**Affiliations:** ^1^ Department of Pharmacy, Faculty of Pharmacy Al‐Zaytoonah University of Jordan Amman Jordan; ^2^ Department of Clinical Pharmacy, Faculty of Pharmacy Jordan University of Science and Technology Irbid Jordan; ^3^ Department of Psychology, School of Social Sciences, Humanities and Law Teesside University Borough Road Middlesbrough UK

**Keywords:** health literacy, Jordan, medication adherence, medication beliefs, multiple sclerosis

## Abstract

**Background and Aim:**

Multiple sclerosis (MS) is a chronic autoimmune disease that affects the central nervous system, often leading to cognitive and physical impairments. Medication adherence is crucial for managing MS, yet nonadherence remains a significant issue, influenced by factors such as health literacy (HL) and beliefs about medication. This study aimed to evaluate HL, medication adherence, and the association between them among patients with MS in Jordan.

**Design:**

A cross‐sectional study was conducted.

**Methods:**

A total of 307 patients with MS from Al‐Bashir Hospital in Amman, Jordan, participated in this study. Patients completed questionnaires assessing HL (HLS‐Q12), medication beliefs (BMQ‐Specific), the Communication and Language Assessment for Multiple Sclerosis (CALMS), and Medication Adherence Report Scale (MARS‐5). Hierarchical logistic regression was conducted to evaluate associations between HL, medication beliefs, and adherence. The study was reported in accordance with the STROBE guidelines for cross‐sectional studies.

**Results:**

The median MARS‐5 score was 25 (IQR: 21–25), indicating high adherence, with 77.5% of participants reporting never skipping doses. Higher HLS‐Q12 scores were significantly associated with better adherence. Receiving medications with less frequent dosing (i.e., monthly or annually administered injectables) was associated with higher adherence compared to daily dosing regimens (i.e., oral medications). Medication necessity beliefs scores were positively associated with medication adherence.

**Conclusion:**

HL, dosage regimen and medication beliefs are significantly associated with adherence among patients with MS in Jordan. Tailored interventions to enhance HL, and promote positive beliefs about medication are important for supporting adherence and health outcomes in this population.

**Abbreviations**
BMQ‐Specific: beliefs about medication questionnaire
CALMS: Communication and Language Assessment for Multiple Sclerosis
CFA: Confirmatory Factor Analysis
CFI: Comparative Fit Index
DMT: disease‐modifying therapy
EPV: Events Per Variable
HL: health literacy
HLS‐Q12: Health Literacy Survey‐12
JD: Jordanian dinars
MARS‐5: Medication Adherence Report Scale
MS: Multiple sclerosis
OR: Odds ratio
PaPA: Perceptions and Practicalities Approach
PIRA: progression independent of relapse activity
RMSEA: Root Mean Square Error of Approximation
SPSS: Statistical Package for the Social Sciences
STROBE: Strengthening the Reporting of Observational Studies in Epidemiology
VIF: Variance Inflation Factors
WHO: World Health Organization

## 1 Introduction

Multiple sclerosis (MS) is a chronic immune‐mediated disease of the central nervous system that causes demyelination and neurodegeneration, leading to a broad spectrum of neurological symptoms and functional impairments [1, 2]. While symptoms can include weakness, sensory disturbances, visual impairment, and cognitive dysfunction, MS is increasingly recognized as a condition where irreversible disability can accrue insidiously, even in the absence of overt relapses, through mechanisms such as progression independent of relapse activity (PIRA). This evolving understanding has intensified the clinical emphasis on early and continuous disease‐modifying therapy (DMT) and the need for patients to develop a complex appreciation of treatment necessity, adherence, and monitoring, even when symptoms are minimal or absent [3, 4].

Therapeutic adherence in MS is therefore a multifaceted phenomenon shaped by patients’ perceptions of disease activity, beliefs about the necessity of treatment, concerns about side effects, and practical challenges associated with chronic medication use [5, 6]. The World Health Organization (WHO) identifies five categories of factors that influence therapeutic adherence: condition‐related factors (e.g., level of disability), therapy‐related factors (e.g., treatment duration), patient‐related factors (e.g., medication beliefs or psychological characteristics), and social and economic factors (e.g., age or education) [7]. Patient adherence to medication remains a big challenge with low patients’ medical adherence reported for various medical conditions [8, 9, 10]. This nonadherence can result in increased illness, mortality, and healthcare expenses. Factors associated with adherence include patients’ beliefs about the importance of medication, their perceived risks and benefits, and practical challenges such as side effects and costs [11].

Identifying factors that are associated with therapeutic adherence in MS is crucial for developing interventions that improve health outcomes. Research has shown that patients with MS who adhere to their treatment tend to share several characteristics, including age, cognitive functioning, levels of depression, satisfaction with treatment, illness duration, symptom severity, presence of comorbidities, method of medication administration, perceived treatment efficacy, and experiences of adverse drug effects [12]. Al‐Keilani (2023) reported that adherence among Jordanian MS patients was associated with gender, education level, and route of medication administration [13]

The Necessity‐Concerns Framework [6] proposes that patients’ adherence is influenced by a personal assessment of the necessity of medication for maintaining health versus concerns about potential adverse effects. In recent years, this model has been refined into the Perceptions and Practicalities Approach (PaPA), which distinguishes between perceptual factors (such as beliefs about medications) and practical factors (such as regimen complexity or memory) that can each facilitate or hinder medication adherence [5]. The PaPA framework emphasizes that effective interventions must address both the perceptual and practical barriers to optimal medication use. In the current study, we assessed both perceptual (necessity, concerns) and practical (dosing frequency) factors, enabling a more comprehensive examination of adherence determinants among patients with MS.

Beliefs about medication play a crucial role in adherence across various patient populations. These beliefs can be categorized into specific necessity beliefs, specific concerns, and general overuse beliefs [14]. Studies have shown that patients with higher necessity beliefs and lower concern or overuse beliefs tend to exhibit higher medication adherence [15]. The Beliefs about Medicines Questionnaire (BMQ) is a valid tool for assessing these beliefs in both medical and psychiatric conditions [16]. Factors such as the severity of depression, cultural background, and migration status can be associated with patients’ beliefs about medications and adherence. Understanding and addressing patients’ beliefs about medications is crucial for supporting adherence, particularly in populations with diverse cultural backgrounds [17]

While both perceptual and practical factors are central to understanding why patients adhere to their medication regimens, these factors do not operate in isolation. An individual's ability to comprehend medical information, navigate the healthcare system, and make informed health decisions may interact with beliefs and practicalities to further shape medication‐taking behavior. Health literacy (HL) has therefore emerged as a crucial construct in efforts to promote adherence and improve clinical outcomes in patients with chronic illnesses such as MS. WHO defines HL as being able to access, understand, appraise, and use information and services in ways that promote and maintain good health and well‐being [18]. HL has been proposed to consist of three types: functional, interactive, and critical HL [19]. HL is vital for managing MS, as it is associated with patients’ understanding of their condition, adherence to treatment, and overall health outcomes. Research indicates that most MS patients have adequate or acceptable HL levels [20]. Inadequate HL levels are associated with more frequent visits to the emergency room and higher rates of hospitalization [21]. Improving HL may be associated with better self‐care, enhanced health promotion, and increased medication adherence, while also decreasing the use of healthcare services [22]. The association between HL and medication adherence has been documented across various chronic conditions, including hypertension [23], diabetes mellitus [24], chronic lower back pain [25], and osteoarthritis [26]. These findings highlight the importance of tailored approaches to assessing and improving HL among diverse populations with chronic illnesses and disabilities [27]. A previous study conducted on the Jordanian population reported inadequate HL, which was associated with advancing age, low educational levels, lack of health insurance, and living in rural areas [28].

In Jordan, the healthcare system consists of public and private sectors, with public hospitals providing subsidized medications for chronic diseases, including MS. However, healthcare access is uneven, with rural populations facing greater challenges in reaching specialized MS clinics [13].

Despite the recognized importance of HL in managing chronic conditions, there is limited evidence regarding HL levels and how they affect medication adherence among patients with MS in Jordan and across the wider Middle East region. Investigating HL and adherence within this population is particularly important, as unique cultural, social, and healthcare system factors are associated with medication‐related behaviors. Understanding these factors can inform targeted interventions to enhance adherence and improve health outcomes. To address this gap, the present study aimed to evaluate HL and medication adherence in patients with MS in Jordan as well as to examine the relationship between them.

### 1.1 Study Hypotheses

Based on previous research and theoretical frameworks such as the Necessity‐Concerns Framework and the PaPA, the study assessed the following hypotheses:

1. HL will be significantly associated with better medication adherence among patients with MS.
2. Stronger necessity beliefs and lower concerns about medications will be associated with higher adherence.
3. Practical factors, specifically less frequent dosing (e.g., monthly or annually administered medications), will be associated with higher adherence compared to daily dosing regimens.

## 2 Methods

### 2.1 Study Design and Participants

This research was reported following the STROBE (Strengthening the Reporting of Observational Studies in Epidemiology) guidelines. The study included 307 patients with MS from Al‐Bashir Hospital in Amman, Jordan, with recruitment taking place between January and May 2024. Eligibility criteria required participants to be Jordanian, at least 18 years old, and to have had a confirmed MS diagnosis for a minimum of 1 year, as documented in their medical records, including neurologists’ notes and previous diagnostic tests. The medical profiles and contact details of eligible patients were retrieved. A trained researcher then contacted all eligible patients by telephone, explained the purpose and procedures of the study, and invited them to participate. This approach ensured that all patients with scheduled appointments at the hospital during the study period who met the inclusion criteria were approached for participation. After the initial telephone contact and explanation, patients who expressed interest in participating were sent a secure electronic link (via WhatsApp or SMS) to complete the informed consent form and the study questionnaire online. The electronic consent form reiterated the study aims, the voluntary nature of participation, and the confidentiality of all collected data. Participants who agreed to take part were required to confirm consent by selecting the option: “I have read the study information, and I agree to participate,” before they could proceed to complete the survey. This method ensured that consent and data collection were both conducted electronically, in accordance with the best practices for remote research during the study period.

### 2.2 Ethics Statement

Ethical approval for the study was granted by the Jordanian Ministry of Health (MOH/REC/2021/263). This research adhered to the ethical principles outlined in the Declaration of Helsinki.

### 2.3 Measures

#### 2.3.1 Medical History and Medications

Patients’ medical history and current medications were retrieved from their medical records. The medications available for patients with MS at Al‐Bashir Hospital are presented in Table A1, in the supplementary material.

#### 2.3.2 Health Literacy

The Arabic version of the Health Literacy Survey‐12 (HLS‐Q12) was used in this study. This instrument is the short version of the European Health Literacy Survey Questionnaire, consisting of 12 items scored on a Likert‐type scale ranging from 1 (“very easy”) to 4 (“very difficult”). The short version of 12 items was validated by [29], demonstrating strong reliability and good model fit. The Arabic translation employed in this study has also been rigorously validated [30], with results demonstrating good internal consistency, achieving a Cronbach's alpha of 0.83. Moreover, a unidimensional structure was confirmed through Confirmatory Factor Analysis (CFA), which yielded strong model fit indices, including a Comparative Fit Index (CFI) of 0.94 and a Root Mean Square Error of Approximation (RMSEA) of 0.058. Further assessment using a Rasch partial credit model revealed high person reliability (0.83) and item separation reliability (0.99), affirming the tool's capacity to effectively differentiate between participant ability levels. The HLS‐Q12 assesses four domains of HL: access, understanding, appraisal, and application. These domains are crucial for accessing and utilizing health information across three key areas: health care, disease prevention, and health promotion.

#### 2.3.3 Beliefs About Medications

The Beliefs about Medicines Questionnaire (BMQ‐Specific) [6, 31] was used in this study. The BMQ‐Specific comprises 10 items, which are divided into two subscales. The Specific‐Concerns scale assesses perceptions of the likelihood of adverse reactions resulting from prescribed medication. The Specific‐Necessity scale addresses patients’ beliefs about their personal need to adhere to prescribed medication. Low scores on the Specific‐Concerns scale suggest fewer concerns about the potential harm of adverse reactions when taking medication regularly, whereas high scores on the Specific‐Necessity scale indicate a strong belief in the necessity of medication for maintaining health. The Arabic version of this scale was validated in a large cohort of Jordanian patients with various chronic diseases, where it demonstrated strong psychometric properties. The validation study confirmed a two‐factor structure representing the Necessity and Concerns subscales. This structure showed excellent internal consistency, with Cronbach's alpha values of 0.89 for the Necessity subscale and 0.93 for the Concerns subscale. Furthermore, CFA indicated a good model fit (CFI = 0.97, RMSEA = 0.07), and both convergent and discriminant validity were established. The BMQ has been widely used in several languages to assess medication beliefs in MS populations [32, 33, 34, 35, 36, 37].

#### 2.3.4 Medication Adherence

The Medication Adherence Report Scale (MARS‐5) was used in the present study to measure participants’ medication adherence. This is a widely used questionnaire designed to assess patients’ adherence to their prescribed medications [31, 38]. It consists of five items with a Likert response format ranging from 1 for “always” to 5 for “never”, where higher scores reflect greater adherence. The Arabic version of the MARS‐5 has been psychometrically validated in a large sample of Jordanian patients with chronic diseases, where it demonstrated a robust, single‐factor structure [31]. The validation study reported excellent internal consistency with a Cronbach's alpha of 0.89. The one‐factor model was further confirmed by CFA, which showed strong model fit indices (CFI = 0.99, RMSEA = 0.08). The MARS‐5 is a well‐established instrument that has been widely used to evaluate medication adherence in MS populations across various international studies, supporting its application in the context of this research [39, 40].

#### 2.3.5 Communication and Language Assessment for Multiple Sclerosis (CALMS)

Language function was evaluated using the Communication and Language Assessment in Multiple Sclerosis (CALMS) [41], a specialized instrument designed for neurological conditions where cognitive and motor deficits can disrupt speech production, comprehension, and pragmatic communication. The scale's 11 items assess key domains commonly affected in MS, including word‐finding, conversational memory, and both expressive and receptive language abilities. In the Arabic version of the questionnaire, participants rate the frequency of these issues on a 3‐point Likert scale (“Never or rarely” to “Usually or always”), with higher scores indicating greater communication difficulty. The Arabic version used in the present study has recently undergone rigorous cultural adaptation and psychometric validation in a Jordanian MS cohort, demonstrating excellent internal consistency (Cronbach's α = 0.93). CFA of the Arabic CALMS supported a unidimensional structure with good model fit (CFI = 0.99, RMSEA = 0.05), and it showed significant correlation with the MSIS‐29, confirming its construct validity (Not yet published).

### 2.4 Sample Size

To determine the appropriate sample size for hierarchical binary logistic regression analysis, the Events Per Variable (EPV) criterion was applied [42]. Adhering to the widely accepted guideline of a minimum of 10 events per predictor variable, the study most complex model with 12 predictors required at least 120 participants in the smaller of the two outcome groups. With 144 patients in the suboptimal adherence group, our study sample comfortably exceeds this requirement, supporting a robust analysis.

### 2.5 Statistical Analysis

Data analysis was performed using the Statistical Package for the Social Sciences (SPSS), version 26. Continuous variables were presented as medians with interquartile ranges (25th –75th percentiles), while categorical variables were presented as frequencies and percentages. The internal consistency of the scales was assessed using Cronbach's alpha. Pearson correlation was applied to assess the correlation between the continuous predictors. Patients were categorized into optimal adherence group (MARS‐5 score = 25) and suboptimal adherence group (MARS‐5 < 25) and a hierarchical binary logistic regression was conducted to assess the associations between various variables and adherence level. The regression model included HLS‐Q12 scores, age, sex, education level, income level, marital status, employment status, dosage frequency, disease duration, CALMS score and Specific‐Necessity and Specific‐Concern scores. Variance Inflation Factors (VIF) were calculated to assess multicollinearity among independent variables. A VIF value greater than 3 was considered indicative of potential multicollinearity. P‐values below 0.05 were considered statistically significant. To evaluate the magnitude of the associations, effect sizes were interpreted using established conventions. For the correlation analyses, the strength of the relationship was interpreted using the correlation coefficient (r) with the following guidelines: small (*r* = 0.10–0.29), medium (*r* = 0.30–0.49), and large (*r* ≥ 0.50). For the hierarchical logistic regression, Odds ratio (OR) and their 95% confidence intervals are reported to describe the magnitude and direction of the association between predictor variables and the median of the medication adherence scores. Moreover, Nagelkerke's Pseudo‐R² was computed to assess model fitness.

## 3 Results

### 3.1 General Demographic Data

Patients’ sociodemographic characteristics are shown in Table 1. The median age was 35 years (interquartile range: 28–44). Most patients were female (68.4%), had completed a bachelor's degree or higher (45%), were married (60.9%), earned less than 500 Jordanian dinars (JD) per month (73.9%), and were unemployed (68.4%).

**Table 1:** Sociodemographic characteristics of the sample (*N* = 307).

Variable		Median (IQR) or frequency (%)
Age		35 (28‐44)
Sex	Female	210 (68.4%)
	Male	97 (31.6%)
Income status	< 500 JDs^a^	227 (73.9%)
	= > 500 JDs	80 (26.1%)
Education level	High school or less	101 (32.9%)
	Diploma	68 (22.1%)
	Bachelor's degree or higher	138 (45%)
Employment status	Unemployed	210 (68.4%)
	Employed	97 (31.6%)
Marital status	Not married	120 (39.1%)
	Married	187 (60.9%)

Illness duration 6 years (3–10).

^a^ Jordanian dinars (1 USD is equivalent to 0.71 JD). The average monthly wage in Jordan is around 500JD (*Average Monthly Wage Reaches JD544 in 2022*, 2024; CEIC, 2025).

### 3.2 Responses to MARS‐5 Items

Patients’ responses to MARS‐5 items are displayed in Table A2 in the appendix. Most patients responded “never” to all five items on the MARS‐5 scale. The item “I change the dose” had the highest frequency of “never” responses (78.2%), followed by “I decide to skip a dose” and “I take medications less than instructed” (77.5%). The item with the lowest frequency of “never” responses was “I forget to take them”, at 60.3%. The median MARS‐5 score was 25 (interquartile range: 21‐25) out of a maximum possible score of 25. The scale demonstrated high internal consistency, with a Cronbach's alpha value of 0.86. Patients on monthly dosing medication had a higher mean MARS‐5 score compared with patients on daily dosing medication (23.06 vs. 21.96) (see Table A3 in the appendix).

### 3.3 Responses to HLS‐Q12 Items

Patients’ responses to HLS‐Q12 items are displayed in Table A4 in the appendix. The majority of patients responded with “difficult” or “very difficult” to most items on the scale. The item “Understand why you need health screenings (e.g. breast exam, blood sugar test, blood pressure)?” received the highest frequency of “very difficult” responses (44%), followed by “Find information on treatments of illnesses that concern you?” (38.1%). Conversely, the item with the highest frequency of “very easy” responses was “Judge if the information in the media on health risks is reliable (TV, internet or other media)?”, at 30.9%. The HLS‐Q12 scale demonstrated acceptable internal consistency, with a Cronbach's alpha value of 0.70. The median score was 35 (interquartile range: 30–48) out of the maximum possible score of 48.

### 3.4 Responses to BMQ Items

Patients’ responses to the BMQ items are displayed in Table A5 in the appendix. Most patients responded with “agree” or “strongly agree” to the items on the Necessity scale. The item “My health, at present, depends on my medicine” had the highest frequency of “strongly agree” responses (44%), while “My medicine protects me from becoming worse” had the highest frequency of “agree” responses (37.1%). On the Concern scale, the item “I sometimes worry about the long‐term effects of my medicine” had the highest frequency of “strongly agree” responses (38.4%), whereas “My medicines disrupt my life” had the highest frequency of “strongly disagree” responses (33.6%). Additionally, “My medicines are mysterious to me” had the highest “neutral” frequency on the Concern scale at 42.7%. The Cronbach's alpha values for the Necessity and Concern scales were 0.85 and 0.65, respectively, indicating good internal consistency for the Necessity scale and moderate consistency for the Concern scale.

### 3.5 Responses to CALMS Items

As shown in Table A6 in the appendix, the highest frequency for the response “Never or rarely” was recorded for the item “Use a lot of vague or empty words such as ‘you know what I mean’ instead of the right word” (31.9%), while the lowest was for “Find it difficult to concentrate enough to understand what is being said?” (18.9%). For the “Sometimes” category, the highest response rate was again observed for the use of vague or empty words (47.6%), whereas the lowest was for “Find it difficult to put ideas together in a logical way?” (34.9%). Under the “Often/Usually or always” category, the highest frequency was for “Find it difficult to remember information on the tip of your tongue?” (42.4%), while the lowest was again for the use of vague or empty words (20.5%). The median score was 12 (7‐18) out of a maximum possible score of 22.

### 3.6 Variables Associated With Medication Adherence

Correlations between continuous predictors of MARS‐5 (Table 2) were assessed and VIF values were computed to identify any multicollinearity and all VIF values were below the cutoff point of 0.3. Hierarchical logistic regression was performed to assess the associations between various variables and MARS‐5 scores (Table 3). In the regression model, higher HLS‐Q12 scores and higher Necessity scores were independently associated with greater odds of being in the optimal adherence group (HLS‐Q12: OR = 1.11, 95% CI 1.05–1.17, *p* < 0.001; Necessity: OR = 1.13, 95% CI 1.06–1.19, *p* < 0.001). These odds ratios are expressed per one‐point increase on the respective scales. Adherence in this cohort was generally high, indicating a ceiling pattern that limits observable variability. Under such conditions, significant predictors can appear with modest unit odds ratios. Moreover, patients receiving daily dosing medications had significantly lower odds of being in the optimal adherence group than those receiving monthly dosing medication (OR = 0.35, 95% CI [0.21–0.60], *p* < 0.001). Finally, the results showed that male participants had significantly higher odds of being in the optimal adherence group compared with females (OR = 2.30, 95% CI [1.29–4.17], *p* = 0.005). The Nagelkerke's Pseudo‐R² indicated that the model explained about 26.5% of the variation in the outcome.

**Table 2:** Correlations between different continuous variables in the model.

	Age	Duration of illness	Necessity score	Concerns score	HLS‐Q12 score	CALMS	Education
Age	1.00						
Duration of illness	0.03	1.00					
Necessity score	0.07	−0.05	1.00				
Concerns score	0.05	0.04	−0.09	1.00			
HLS‐Q12 score	−0.04	0.03	0.04	0.35^a^	1.00		
CALMS score	−0.02	0.03	0.08	−0.34	−0.23	1.00	
Education	−0.08	−0.01	−0.05	0.123^b^	0.06	−0.11	1.00

^a^ *Correlation* is significant at the 0.05 level (2‐tailed).

^b^ *Correlation* is significant at the 0.01 level (2‐tailed).

**Table 3:** Logistic regression of variables associated with MARS‐5 scores.

	Model 1	Model 2	Model 3						
**Characteristic**	**OR**	**95% CI**	***p*‐value**	**OR**	**95% CI**	***p*‐value**	**OR**	**95% CI**	***p*‐value**
Age (years)	1.01	0.99, 1.04	0.3	1.01	0.98, 1.03	0.5	1.01	0.98, 1.03	0.7
Sex (males vs females)	1.59	0.96, 2.68	0.075	1.80	1.06, 3.10	0.032	2.30	1.29, 4.17	0.005**
Employed versus Unemployed	0.69	0.39, 1.21	0.2	0.74	0.42, 1.32	0.3	0.67	0.36, 1.23	0.2
Married versus Unmarried	1.10	0.67, 1.81	0.7	1.12	0.67, 1.88	0.7	1.31	0.76, 2.28	0.3
Education level									
Diploma	0.56	0.30, 1.05	0.074	0.55	0.28, 1.06	0.077	0.55	0.27, 1.11	0.10
Bachelor's degree or higher	0.76	0.42, 1.38	0.4	0.61	0.33, 1.15	0.13	0.69	0.35, 1.35	0.3
High school or less	(Reference group)								
Income status (high vs. low)	1.19	0.67, 2.14	0.6	1.37	0.75, 2.54	0.3	1.00	0.52, 1.94	> 0.9
Duration of illness				1.00	1.00, 1.00	0.8	1.00	1.00, 1.00	0.9
Daily versus monthly dosing				0.35	0.21, 0.56	< 0.001	0.35	0.21, 0.60	< 0.001**
CALMS score				1.02	0.98, 1.06	0.3	1.03	0.99, 1.08	0.14
HLS‐Q12 score							1.11	1.05, 1.17	< 0.001**
Concerns score							1.01	0.95, 1.08	0.8
Necessity score							1.13	1.06, 1.19	< 0.001**

**Abbreviation:** CI = Confidence Interval.

** Significant at the 0.01 level (2‐tailed).

## 4 Discussion

The present study examined the relationship between HL, beliefs about medication, and medication adherence among patients with MS in Jordan. While the association between health literacy, medication beliefs, and adherence has been extensively examined in the broader literature, including in studies involving Arabic‐speaking populations [12], this study is, to our knowledge, the first to assess these relationships specifically in patients with MS in Jordan.

### 4.1 Medication Adherence and Associated Factors

The high medication adherence levels observed in this study are consistent with findings from similar research in healthcare settings. The availability of MS medications exclusively at hospitals and their monthly dosing regimens likely facilitated adherence by reducing logistical barriers. This finding aligns with prior studies demonstrating the role of accessible healthcare systems in supporting adherence [21, 27]. The higher adherence observed among patients receiving monthly/biannually injectable medications may be explained by the hospital protocol, where these treatments are administered directly in the hospital under medical supervision. In these cases, adherence is effectively ensured by the structured nature of the treatment regimen, rather than relying solely on patient behaviour. This highlights the need to distinguish between administration modality (such as oral *vs.* injectable) and dosing schedule (such as daily *vs.* monthly or annual), as both can influence adherence in different ways.

Similar findings have been reported in other studies. For example, Washington and Langdon [12] found that regular contact with healthcare providers, structured treatment follow‐up, and ongoing monitoring were associated with higher adherence to disease‐modifying therapies among patients with MS [12]. These findings suggest that a well‐organized healthcare infrastructure can support adherence by reinforcing routine, addressing patient concerns in real time, and facilitating accountability. This is further supported by previous research among Jordanian patients with MS, which found higher adherence levels in patients receiving injectable medications [13].

It is important to clarify that while some MS therapies, such as natalizumab, require monthly infusions administered in hospital, others, like ofatumumab, are delivered via home injection but still involve regular clinic‐based monitoring. As such, patients on different regimens vary in how frequently they engage with healthcare professionals. This visit frequency may itself influence adherence by providing opportunities for reinforcement, supervision, and early intervention. Research in MS and other chronic conditions has shown that structured treatment regimens and regular follow‐up with providers are associated with better adherence outcomes [43, 44]. Therefore, adherence patterns in our sample may reflect not only medication type and administration route, but also how closely patients are followed up. While biannual review may be appropriate for some clinically stable patients, others, particularly those receiving infusions, require more intensive schedules. These differences should be considered when interpreting our findings.

Some research has found that patients receiving oral therapies reported adherence rates comparable to those on injectable treatments, particularly when oral regimens were perceived as easier to integrate into daily routines and involved fewer side effects [45]. These varied findings point to the influence of multiple factors, including convenience, patient preferences, beliefs about medication effectiveness, and cultural attitudes toward different treatment routes. Further research is needed to better understand how these factors interact, particularly within the Jordanian healthcare context, in order to support patient decision‐making and improve adherence strategies.

Patients in the present study had a median HL score of 35 out of a maximum of 48, which indicates a moderate level of health literacy. While this score is broadly in line with findings from the Arabic validation of the HLS‐Q12 in chronically ill Jordanian patients [30], it appears somewhat lower than those reported in the original European Health Literacy Survey, where mean scores ranged between 32.9 and 38.2 across countries using the longer HLS‐EU‐Q47 [29]. However, direct comparisons should be interpreted with caution due to differences in tools and populations.

Despite this, the significant proportion who reported finding key tasks, such as understanding health screenings, “difficult” or “very difficult” highlights important gaps in functional HL that may affect their ability to engage effectively with healthcare services. These findings align with global research, which suggests that while many patients with MS demonstrate adequate overall HL, specific challenges persist in areas such as accessing, appraising, and applying health information [45]. This points to the importance of developing focused educational support to help patients strengthen these particular areas, especially in healthcare settings where navigating services can be more challenging.

This study also found that patients’ beliefs about their medication were significantly associated with adherence. Specifically, participants with stronger beliefs about the necessity of their medication and fewer concerns about negative effects were more likely to adhere to their prescribed regimens. These findings are consistent with the Necessity‐Concerns Framework [6], which posits that patients balance their perceived need for treatment against their concerns about potential harms. Similar patterns have been observed in the region. For example, Al‐Keilani and Almomani [13] reported that medication beliefs were a key determinant of adherence among Jordanian patients with MS [13]. Alhazzani et al. [44] also found that beliefs and treatment satisfaction influenced adherence in a Saudi MS population [44]. Together, these findings emphasize the importance of addressing patients’ medication beliefs within culturally specific healthcare contexts to improve adherence outcomes.

The positive association between HL and adherence observed in this study is consistent with a recent systematic review that documented similar findings across chronic diseases, including diabetes and hypertension [46]. However, differences in demographic profiles between MS and other chronic condition populations may influence the generalizability of these comparisons. Further research directly comparing these groups would help clarify how sociodemographic characteristics, including education and HL levels, shape adherence across conditions.

Higher HL equips patients with the skills needed to understand their diagnosis, interpret medical information, evaluate treatment options, and follow complex medication regimens; factors that are particularly relevant in managing long‐term conditions such as MS. In contrast, limited HL can hinder a patient's ability to engage with health services, leading to misunderstandings about treatment, reduced adherence, and ultimately poorer health outcomes. This has been well documented, with evidence linking inadequate HL to increased hospitalisations and diminished health status [21]. These findings highlight the value of regularly assessing HL in clinical practice and adapting communication approaches to meet patients’ individual needs and understanding.

The strong association between necessity beliefs and dosage frequency and higher adherence scores supports the Necessity‐Concerns Model [6] and the more recent PaPA [5], which emphasizes that both patients’ beliefs about their medications and practical aspects of medication‐taking, such as dosing frequency, contribute to adherence. This association between stronger beliefs in medication necessity and higher adherence has also been observed in other chronic conditions. For instance, studies of patients with diabetes have shown that those who perceive their medication as essential and express fewer concerns about side effects or dependency are more likely to adhere to treatment regimens [47, 48]. These findings emphasise the importance of addressing patients’ beliefs during clinical consultations, by reinforcing the benefits and necessity of treatment, as well as openly discussing and mitigating concerns that could act as barriers to adherence.

As shown in the regression, health literacy and medication‐related beliefs contribute to adherence, but their effects are detected at the level of single scale points. Because both instruments have a relatively wide range, clinically relevant change will usually occur over several points, so the effect of the predictors should be regarded as cumulative rather than as a single‐point shift. As scores improve across several points, the effect becomes larger than it appears from the unit OR. For example, a 3‐point improvement in health literacy corresponds to 1.11³ ≈ 1.37 ( ≈ 37% higher odds), and a 3‐point improvement in necessity corresponds to 1.13³ ≈ 1.44 ( ≈ 44% higher odds). These examples show that small point‐level effects can accumulate into clinically meaningful gains when patients achieve multi‐item improvements, even though the unit odds ratios appear modest. At the same time, adherence scores in this sample were clustered near the upper end, which attenuates detectable associations and helps explain why statistically robust predictors yielded odds ratios around 1.1. The Nagelkerke pseudo‐R² of 26.5% is therefore consistent with a model that captures important modifiable factors (literacy, necessity beliefs, regimen complexity, sex) within a population that is already highly adherent.

### 4.2 Implications for Practice

The findings of the current study highlight the critical need for healthcare providers to integrate HL assessments and evaluations of medication beliefs into routine care for patients with MS in Jordan and the broader Middle East region. Tailored interventions aimed at improving HL, particularly in areas identified as challenging, such as understanding medical information and appraising treatment options, should be prioritized. For instance, using patient‐friendly educational materials that incorporate visuals, plain language, and culturally appropriate examples can help bridge comprehension gaps. Research has shown that interventions designed to simplify communication, such as using plain language and visual aids, can significantly enhance patients’ ability to comprehend and act on health information [49]. Additionally, addressing the challenges of shared decision‐making among patients with lower literacy by developing appropriate frameworks can further support patient engagement [50]. These approaches ensure that HL interventions are accessible and relevant, maximizing their impact on patient outcomes.

Digital tools, such as mobile applications and web‐based platforms, have demonstrated potential in enhancing medication adherence and HL. For instance, mobile health applications can provide medication reminders, educational content, and real‐time feedback, serving as valuable resources for patients. A systematic review and meta‐analysis [51] found that diabetes self‐management education and digital support applications significantly improved medication adherence and clinical outcomes in patients with type 2 diabetes mellitus.

Training healthcare providers in HL‐sensitive communication techniques and medication adherence strategies is crucial for effectively identifying and addressing barriers to adherence. Evidence indicates that such training enhances patient outcomes. For instance, a randomized controlled trial demonstrated that physicians who received communication skills training significantly improved their patients’ HL, self‐efficacy, medication adherence, and blood pressure control compared to a control group [52]. Additionally, equipping healthcare professionals with HL‐sensitive communication skills enables them to tailor their approaches to individual patient needs, thereby improving medication adherence and overall health outcomes [53].

### 4.3 Limitations and Future Directions

The present study was conducted at a single public hospital, Al‐Bashir Hospital, in Amman, Jordan. While this institution is one of the largest in the country and serves a socioeconomically and culturally diverse patient population, the single‐center design limits the generalizability of the findings to other healthcare settings, regions, or countries. Future research should aim to include multiple centers, both urban and rural, to capture a broader spectrum of healthcare environments and patient demographics. This approach would provide a more comprehensive understanding of adherence and HL across different populations and healthcare systems.

Another limitation of the study is that the BMQ primarily assesses intentional non‐adherence, reflecting patients’ conscious decisions based on their beliefs about the necessity and potential harm of medications. According to Horne's model [6], this captures only part of the adherence spectrum, as unintentional non‐adherence, such as forgetting doses or misunderstanding instructions, is influenced by different factors, including cognitive capacity, routine, and healthcare communication. Therefore, while our findings shed light on belief‐related drivers of adherence, they may underestimate the role of unintentional factors. Future research could use complementary tools to assess both forms of non‐adherence more comprehensively.

A further limitation is the cross‐sectional nature of the study, which precludes the ability to establish causal relationships between HL, beliefs about medicines, and medication adherence. Longitudinal studies are needed to track changes in HL and adherence behaviours over time, especially considering the progressive nature of MS and the potential influence of treatment fatigue or evolving beliefs about medication.

Additionally, while the tools used in this study were validated and reliable, self‐reported measures of adherence and HL may be subject to social desirability bias or recall bias. Future research could incorporate objective measures of adherence, such as pharmacy refill data or electronic medication monitoring, to complement self‐reported data and provide a more accurate assessment.

The study did not account for certain potentially influential variables, such as psychological factors (e.g., depression or anxiety), which are known to affect adherence and HL in chronic disease populations [54, 55]. Including these factors in future research could provide a more holistic view of the determinants of adherence in MS patients.

Although the CALMS questionnaire provides valuable insight into self‐perceived communication and language difficulties among MS patients, which can be important contributors to both HL and medication adherence, it remains is a patient‐reported outcome and does not capture the broader range of cognitive impairments, such as memory or processing speed, commonly observed in MS. Furthermore, the reliability of self‐report may be limited by participants’ insight or awareness of their difficulties, particularly in those with cognitive impairment. Despite these limitations, the inclusion of CALMS offers a relevant perspective on communication‐related barriers, but findings should be interpreted within the context of its scope. Future research should supplement self‐report tools like CALMS with objective cognitive assessments to better elucidate the impact of cognitive function on HL and adherence.

Finally, while this study focused on patients in Jordan, there is a lack of comparative research exploring adherence and HL in MS patients across Middle Eastern countries. Comparative studies could highlight regional similarities and differences to support the design of culturally tailored interventions.

## 5 Conclusion

This study highlights the interconnected roles of HL, beliefs about medication, and adherence in patients with MS in Jordan, providing valuable insights into the challenges and opportunities within this population. The findings demonstrate that higher HL levels and stronger necessity beliefs about medications are positively associated with adherence, while longer disease duration poses a challenge. Our findings point to a critical need for tailored interventions that improve HL, address patient concerns, and sustain adherence over the long term. Future research should build on these findings by expanding to diverse settings, employing longitudinal designs to explore changes in HL and adherence over time, and testing the effectiveness of targeted interventions. Adopting a multidisciplinary and patient‐centred approach may help healthcare systems better meet the needs of patients with MS.

## Author Contributions

Conceptualization, Walid Al‐Qerem and Sawsan Khdair; methodology, Walid Al‐Qerem; software, Walid Al‐Qerem; validation, Judith Eberhardt and Anan Jarab; formal analysis, Walid Al‐Qerem, Anan Jarab, Sawsan Khdair and Dunia Basem; investigation, Sawsan Khdair and Dunia Basem; data curation, Walid Al‐Qerem and Dunia Basem; writing, original draft preparation, Walid Al‐Qerem, Anan Jarab, Sawsan Khdair, Dunia Basem, and Judith Eberhardt; writing, review and editing, Walid Al‐Qerem, Anan Jarab, Sawsan Khdair, Dunia Basem, and Judith Eberhardt; visualization, Walid Al‐Qerem; supervision, Walid Al‐Qerem; project administration, Walid Al‐Qerem All authors have read and agreed to the published version of the manuscript.

## Funding Information

This research was conducted without any external funding or financial support.

## Ethics Statement

The present study received ethical approval from the Jordanian Ministry of Health (MOH/REC/2021/263). All participants were informed about the purpose of the study, its potential benefits and risks, and their rights as participants, and written informed consent was obtained prior to participation.

## Conflicts of Interest

The author(s) report no conflicts of interest in this work. All authors have read and approved the final version of the manuscript. Judith Eberhardt had full access to all of the data in this study and takes complete responsibility for the integrity of the data and the accuracy of the data analysis.

## Consent

All participants provided informed consent prior to taking part in the study. They were fully informed about the study's purpose, procedures, potential risks and benefits, and their right to withdraw at any time without consequences to their care.

## Transparency Statement

The lead author, Judith Eberhardt, affirms that this manuscript is an honest, accurate, and transparent account of the study being reported; that no important aspects of the study have been omitted; and that any discrepancies from the study as planned (and, if relevant, registered) have been explained.

## Supporting information

Table A1 ‐ Supplementary Material (1).

## Data Availability

The dataset supporting the findings of this study is publicly available in Zenodo: Al‐Qerem, W. (2025). Adherence among MS patients [Data set]. Zenodo. https://doi.org/10.5281/zenodo.15073308
