# Supplementary material for: Health Literacy, Beliefs About Medication, and Medication Adherence in Patients With Multiple Sclerosis in Jordan: A Cross‐Sectional Study
Source: Health Sci Rep. 2025 Dec 18;8(12):e71672. doi: 10.1002/hsr2.71672 (PMC12715330; doi:10.1002/hsr2.71672)
Supplement: Supplementary file 1 — Table A1 ‐ Supplementary Material (1). [file HSR2-8-e71672-s001.docx]

Table A1. Available medications for MS patients in Al-Basher Hospital

| Medication | Route of administration | Dosage frequency | Frequency (%) |
| --- | --- | --- | --- |
| Dimethyl fumarate tab | oral | Twice a day | 101 (32.9%) |
| Fingolimod 0.5 mg | oral | Once a day | 53 (17.3%) |
| Natalizumab 300 mg/15ml | infusion | Once a month | 16 (5.2%) |
| Ocrelizumab 300 mg /15ml | infusion | Twice a year | 31 (10.1%) |
| Ofatumumab 20 mg | subcutaneously | Once a month | 124 (40.4%) |

Table A2 Patients’ responses to MARS-5 items (Frequency [%])

|  | Always | Often | Sometimes | | Rarely | Never |
| --- | --- | --- | --- | --- | --- | --- |
| I forget to take them | 9 (2.9%) | 7 (2.3%) | 44 (14.3%) | | 62 (20.2%) | 185 (60.3%) |
| I change the dose | 3 (1%) | 9 (2.9%) | 26 (8.5%) | | 29 (9.4%) | 240 (78.2%) |
| I stop taking them for a while | 10 (3.3%) | 7 (2.3%) | 32 (10.4%) | | 32 (10.4%) | 226 (73.6%) |
| I decide to skip a dose | 4 (1.3%) | 10 (3.3%) | 27 (8.8%) | | 28 (9.1%) | 238 (77.5%) |
| I take medications less than instructed | 8 (2.6%) | 8 (2.6%) | 24 (7.8%) | | 29 (9.4%) | 238 (77.5%) |
| Adherence state | Optimal adherence | | | 163 (53.1%) | | |
|  | Suboptimal adherence | | | 144 (46.9%) | | |

Table A3. MARS 5 score in patients receiving Annual vs. Daily/Monthly dosing, Mean (SD)

| Dose regimen | Mean (SD) |
| --- | --- |
| Monthly dosing medication | 23.06 (3.56) |
| Daily dosing medication | 21.96 (3.87) |

Table A4. Patients' responses to HLS-Q12 items (Frequency [%])

| Items | Very Easy | Easy | Difficult | Very Difficult |
| --- | --- | --- | --- | --- |
| find information on treatments of illnesses that concern you? | 83 (27%) | 56 (18.2%) | 51 (16.6%) | 117 (38.1%) |
| to understand information about what to do in a medical emergency? | 35 (11.4%) | 72 (23.5%) | 117 (38.1%) | 83 (27%) |
| to judge the advantages and disadvantages of different treatment options? | 38 (12.4%) | 68 (22.1%) | 118 (38.4%) | 83 (27%) |
| follow the instructions on medication? | 49 (16%) | 19 (6.2%) | 129 (42%) | 110 (35.8%) |
| Find information on how to manage mental health problems like stress and depression? | 69 (22.5%) | 75 (24.4%) | 105 (34.2%) | 58 (18.9%) |
| understand why you need health screenings (e.g. breast exam, blood sugar test, blood pressure)? | 23 (7.5%) | 30 (9.8%) | 119 (38.8%) | 135 (44%) |
| judge if the information in the media on health risks is reliable (TV, internet or other media)? | 95 (30.9%) | 63 (20.5%) | 117 (38.1%) | 32 (10.4%) |
| decide how you can protect yourself from illness based on advice from family and friends? | 37 (12.1%) | 46 (15%) | 131 (42.7%) | 93 (30.3%) |
| Find information on health attitudes such as exercise, healthy food and nutrition? | 33 (10.7%) | 36 (11.7%) | 143 (46.6%) | 95 (30.9%) |
| Understand information on food packaging? | 61 (19.9%) | 53 (17.3%) | 139 (45.3%) | 54 (17.6%) |
| judge which everyday behaviour is related to your health (drinking and eating habits, exercise etc.)? | 20 (6.5%) | 51 (16.6%) | 133 (43.3%) | 103 (33.6%) |
| to make decisions to improve your health and well-being? | 26 (8.5%) | 60 (19.5%) | 114 (37.1%) | 107 (34.9%) |

Table A5. Patients’ responses to BMQ items (Frequency (%))

|  | Strongly disagree | Disagree | Neutral | Agree | Strongly agree |
| --- | --- | --- | --- | --- | --- |
| Necessity Scale | | | | | |
| My health, at present, depends on my medicine | 22 (7.2%) | 14 (4.6%) | 54 (17.6%) | 82 (26.7%) | 135 (44%) |
| My medicine protects me from becoming worse | 14 (4.6%) | 9 (2.9%) | 39 (12.7%) | 114 (37.1%) | 131 (42.7%) |
| My health in future will depend on my medicine | 26 (8.5%) | 14 (4.6%) | 137 (44.6%) | 87 (28.3%) | 43 (14%) |
| My life would be impossible without my medicine | 53 (17.3%) | 42 (13.7%) | 75 (24.4%) | 64 (20.8%) | 73 (23.8%) |
| Without my medicine, I would be very sick | 28 (9.1%) | 29 (9.4%) | 71 (23.1%) | 68 (22.1%) | 111 (36.2%) |
| Concerns scale | | | | | |
| Having to take medicine worries me | 34 (11.1%) | 63 (20.5%) | 63 (20.5%) | 65 (21.2%) | 82 (26.7%) |
| I sometimes worry about the long-term effects of my medicine | 23 (7.5%) | 30 (9.8%) | 36 (11.7%) | 100 (32.6%) | 118 (38.4%) |
| My medicines are mysterious to me | 28 (9.1%) | 62 (20.2%) | 131 (42.7%) | 49 (16%) | 37 (12.1%) |
| My medicines disrupt my life | 103 (33.6%) | 116 (37.8%) | 51 (16.6%) | 23 (7.5%) | 14 (4.6%) |
| I sometimes worry about becoming too dependent on my medicine | 26 (8.5%) | 39 (12.7%) | 54 (17.6%) | 80 (26.1%) | 108 (35.2%) |
| This medicine gives me unfavorable side effects | 73 (23.8%) | 75 (24.4%) | 49 (16%) | 61 (19.9%) | 49 (16%) |

Table A6. Reordered Frequencies (%) of the Communication and Language Assessment questionnaire for Multiple Sclerosis (CALMS)

| Item | Never or rarely | Sometimes | Often/Usually or always |
| --- | --- | --- | --- |
| Have difficulty thinking of the particular word you want? | 69 (22.5%) | 127 (41.4%) | 111 (36.1%) |
| Have difficulty remembering your train of thought as you are speaking? | 68 (22.1%) | 119 (38.8%) | 120 (39.1%) |
| Use a lot of vague or empty words such as “you know what I mean” instead of the right word? | 98 (31.9%) | 146 (47.6%) | 63 (20.5%) |
| Find it difficult to remember information on the tip of your tongue? | 59 (19.2%) | 118 (38.4%) | 130 (42.4%) |
| Find it difficult to convey precisely what you mean? | 69 (22.5%) | 120 (39.1%) | 118 (38.4%) |
| Leave out important details? | 83 (27%) | 126 (41%) | 98 (31.9%) |
| Find it difficult to concentrate enough to understand what is being said? | 58 (18.9%) | 123 (40.1%) | 126 (41%) |
| Find it difficult to remember recent conversations? | 69 (22.5%) | 110 (35.8%) | 128 (41.7%) |
| Find it difficult to keep track of the main details of conversations? | 88 (28.7%) | 118 (38.4%) | 101 (32.9%) |
| Find it difficult to put ideas together in a logical way? | 80 (26.1%) | 107 (34.9%) | 120 (39.1%) |
| Hesitate, pause, or repeat yourself? | 80 (26.1%) | 122 (39.7%) | 105(34.2%) |
